# Tinnitus and associations with chronic pain: The population-based Tromsø Study (2015–2016)

**DOI:** 10.1371/journal.pone.0247880

**Published:** 2021-03-02

**Authors:** Jannike H-L Ausland, Bo Engdahl, Bente Oftedal, Ólöf A. Steingrímsdóttir, Christopher S. Nielsen, Laila A. Hopstock, Magnar Johnsen, Oddgeir Friborg, Jan H. Rosenvinge, Anne E. Eggen, Norun H. Krog

**Affiliations:** 1 Department of Environmental Health, Norwegian Institute of Public Health, Skøyen, Oslo, Norway; 2 Department of Chronic Diseases and Ageing, Norwegian Institute of Public Health, Skøyen, Oslo, Norway; 3 Department of Pain Management and Research, Oslo University Hospital, Nydalen, Oslo, Norway; 4 Department of Community Medicine, UiT The Arctic University of Norway, Langnes, Tromsø, Norway; 5 Department of Occupational and Environmental Medicine, University Hospital of North Norway, Tromsø, Norway; 6 Department of Psychology, UiT The Arctic University of Norway, Langnes, Tromsø, Norway; Universiteit Antwerpen, BELGIUM

## Abstract

Tinnitus and pain have many similarities. Both are subjective sensations that may turn chronic, they are often accompanied by hypersensitivity in their respective sensory system, and overlapping brain changes have been observed. Since no population study has examined the empirical association between chronic pain and tinnitus, the present study aimed to explore the relationship in a general adult population. We used data from the seventh survey of the Tromsø Study (2015–2016). Participants (aged ≥40) responded to questions about pain and tinnitus. Using multiple logistic regression, we analysed the adjusted relationship between chronic pain and tinnitus in the full sample (n = 19,039), using several tinnitus definitions ranging from tinnitus >5 minutes within the past 12 months (broadest definition) to at least weekly and highly bothersome tinnitus (strictest definition). We also analysed relationships between number of body regions with pain, pain intensity and bothering, and tinnitus >5 minutes, among participants with chronic pain (n = 11,589). We found an association between chronic pain and tinnitus that was present irrespective of tinnitus definition, but was stronger with more bothersome tinnitus. With chronic pain, the odds of tinnitus >5 minutes was 64% higher, while odds of at least weekly, highly bothersome tinnitus was 144% higher than without chronic pain. Among participants with chronic pain, the number of pain regions was the pain variable most strongly associated with tinnitus >5 minutes (OR = 1.17 (95% CI: 1.14–1.20) for an increase of one region), whereas the other pain variables (intensity and bothering) showed weaker associations. All chronic pain variables had significant interactions with age, with the strongest associations for the youngest individuals (40–54 years). Our findings support the existence of an association between chronic pain and tinnitus and emphasises the importance of examining for comorbid pain in tinnitus patients to provide a more comprehensive treatment of tinnitus.

## Introduction

Tinnitus is a common condition that can be severely debilitating. It is clinically defined as the sensation of sound in the absence of external stimulation [1], and international studies indicate a prevalence of 5–43% in the general population [2], depending on how it is operationally defined. Tinnitus has been linked to various health problems [3]. Among these are several pain syndromes, such as headache [4], temporomandibular disorder [5], and fibromyalgia [6]. To our knowledge, no population study of the general tinnitus–chronic pain relation has been performed. Current knowledge is based on small clinical studies [7] or studies of specific pain syndromes [4–6], and these results are not necessarily valid for the general population. A study of tinnitus in patients with chronic pain reported a high prevalence of tinnitus, with more than half of the participants experiencing the disorder (54%, n = 72) [7]. Another study found associations between tinnitus-related distress and pain experience in patients with chronic tinnitus (n = 1,238) [8]. However, empirical evidence of a tinnitus–pain association is still scarce, and knowledge on the topic remains fragmented, although thoroughly theoretically discussed [1,9–11]. Several authors have pointed out similarities between tinnitus and pain. Both are subjective sensations [1], may develop into chronic conditions [9], are often accompanied by hypersensitivity to sensory stimuli [10], and frequently co-occur with anxiety and depression [12].

Similar underlying neurological mechanisms have been proposed for the two conditions. It appears that both chronic tinnitus and chronic pain are caused by functional changes in parts of the central nervous system, expressed through synaptic plasticity. These changes may be triggered by damage to peripheral structures (i.e. ear/skin area) [10] and manifested through deafferentation of nerve fibres [1]. Furthermore, similar changes in brain structure and activity have been observed in tinnitus and chronic pain, involving higher-order brain regions with cognitive and affective functions [11].

Hyperacusis, decreased tolerance to ordinary environmental sounds, appears to be common in chronic pain syndromes. Hence, it has been suggested that the auditory and nociceptive systems are connected at convergence sites in the brain, possibly causing cross-modal activation, such that hyperactivity spreads from one system to the other [13]. This is supported by the finding that tinnitus may be triggered or modified through other sensory modalities than the auditory system [14]. A shared hyperresponsive network between hyperacusis and hyperalgesia has also been suggested [15]. Taken together, these findings point towards a direct link between the auditory and pain systems, making pain a likely comorbidity with tinnitus. Since both tinnitus and pain can be accompanied by hypersensitivity to stimuli in their respective sensory system, a possible comorbidity between the two conditions may be caused by an underlying generalised hypersensitivity to sensory stimuli.

Tinnitus and chronic pain are both frequently occurring phenomena that vary immensely in characteristics and symptom intensity. If the auditory and nociceptive systems are in fact connected, one might expect that tinnitus and chronic pain are associated irrespective of the severity and anatomic location of pain–that is, a more general association between the two phenomena. Still, as indicated by a study on chronic shoulder pain, osteoarthritis and fibromyalgia among tinnitus patients, the degree of tinnitus bother may affect the strength of the association [16].

The present study examines the relationship between chronic pain and tinnitus in a large general adult population. We look at the association at a general level by examining perceived symptoms of both phenomena irrespective of their original cause, to capture the whole range of tinnitus and chronic pain in the population. We investigate if the association differs with the severity of tinnitus, and assess the importance of number of body regions with chronic pain, pain intensity, and bothering in the association between tinnitus and chronic pain. We also examine the potential moderating role of sex, age, worry, and noise sensitivity in the relationships.

## Methods

### Study sample

We used data from the seventh survey of the population-based Tromsø Study (Tromsø 7, 2015–2016), conducted in the municipality of Tromsø, Norway. This study has been run since 1974, inviting both whole birth cohorts and random samples [17]. It started out as a study that focused on cardiovascular disease, but is now a comprehensive health study that includes research on e.g. cancer, lung disease, diabetes, and mental health [18]. In Tromsø 7, questions on tinnitus were included for the first time. All inhabitants in the municipality of Tromsø aged 40 and above (n = 32,591) were invited to participate in Tromsø 7. In total, 21,083 people aged 40–99 (65%) participated.

All participants completed biological sampling, clinical examinations, interviews, and answered electronic questionnaires including questions on pain and tinnitus. Due to a delay in the roll-out of the chronic pain questionnaire, 820 participants did not receive the questionnaire and were excluded from the sample. Of the remaining sample, data for one or more study variables was missing for 1,224 subjects, leaving a sample of 19,039 for statistical analyses. The amount of missing for individual variables ranged from 0–2.9%.

A detailed overview of the data collection process, including links to the main questionnaires, can be found on the website of the Tromsø Study (English: https://uit.no/research/tromsostudy/project?pid=708909 / Norwegian: https://uit.no/research/tromsoundersokelsen/project?pid=706786). Details about the Tromsø Study can also be found in previously published articles [17,19].

The study was approved in writing by the Regional Ethical Committee of Northern Norway.

### Main variables

#### Tinnitus

Participants were asked about tinnitus in a questionnaire: “During the last 12 months, have you experienced ringing in your ears lasting more than five minutes?” (yes/no). “Tinnitus lasting more than five minutes” is the most commonly used operational definition in population studies [2], and to have experienced this during the past year has more recently been suggested as the standard way of measuring tinnitus prevalence in such studies [20].

Respondents who answered “yes” to the above question were further asked about the frequency of their tinnitus: “How frequently do you have ringing in your ears?” (less frequently than every week / each week, but not every day / each day, but not all the time / most of the time). They were also asked to rate the degree to which it bothered them: “Please, indicate how bothered you are by the ringing in your ears?” (numeric rating scale 0–10, ranging from «not bothered» to “worst imaginable bother”). These two questions were based on questions that were included in the Nord-Trøndelag Hearing Loss Study, part of the Nord-Trøndelag Health Study (HUNT-2) (https://www.ntnu.edu/hunt). However, instead of using the three categories used to rate “bother” in HUNT-2, which is a rather crude measure, it was chosen to use an eleven-point bother scale that has been tested in other contexts [21], and also was used for the ratings of pain intensity and bother in the Tromsø Study. We have not found studies aimed at validating the tinnitus questions included, but the selection of tinnitus variables was based on questions used before in other studies and were thus pre-tested.

In our main analyses, we used both a wide tinnitus definition, to capture the general association between chronic pain and tinnitus, as well as tinnitus definitions restricted to frequent and bothersome tinnitus. Hence, we first looked at the association between chronic pain and any tinnitus, i.e. tinnitus lasting more than five minutes within the past 12 months, a variable hereby referred to as “tinnitus >5 minutes”. Then, we included the variables for tinnitus frequency and bother in the tinnitus definitions.

Our stricter tinnitus definitions included participants with at least weekly tinnitus, in line with findings from a study of tinnitus in children, where researchers found that tinnitus was more likely to be clinically significant if it occurred more than once a week [22]. Additionally, we used three separate bother levels; (at least) a little bothered (bother ≥3), bothered (bother ≥6), and highly bothered (bother ≥8). This is in accordance with cut-offs that are used for a similar annoyance scale to measure noise annoyance from external sources [21,23]. As a result, our analyses included one definition containing everyone with tinnitus, and three tinnitus definitions restricted by frequency and increasing degrees of bother.

#### Chronic pain

The Graphical Index of Pain (GRIP) was used to collect information about pain location, pain distribution, and pain characteristics [24]. GRIP is a hierarchical body map structured in two tiers. In the first tier, participants were presented with pictures of the human body (front and back), divided into ten main regions, and were asked to mark the region(s) where they had experienced pain during the last four weeks (except transient short-term pain and menstrual pain). For each marked first tier region, the participants also answered a battery of questions about pain characteristics, including time since onset (less than 4 weeks/1–2 months/3–5 months/6–11 months/1–2 years/3–5 years/more than 5 years with information about age at pain onset), pain intensity last four weeks (How strong has the pain usually been?) and bothering (How much has the pain usually bothered you?) during the last four weeks. Both pain intensity and bothering were measured on numeric rating scales (0–10, ranging from “no pain” to “the strongest imaginable pain” and “no bother” to “the greatest imaginable bother”, respectively). For each marked first tier region, participants were presented with a second tier detailing pain location within the first tier region. Since data from the second tier were not included in our analyses, these will not be described further. GRIP was pre-tested among patients in a physiotherapy clinic and healthy volunteers before it was applied in the Tromsø Study. For further descriptions of GRIP, we refer to Steingrímsdóttir et al. [24].

We defined chronic pain as pain that had been present for three months or longer, in accordance with the definition of the World Health Organization [25]. It should be noted that chronic pain has been operationally defined in a variety of ways in pain epidemiology [26]. Here, an individual was considered to have chronic pain if they had marked at least one of the first tier body regions and the pain had lasted at least three months. For participants with chronic pain in one or more body regions, three different measures of pain severity were used in the analyses: 1) Number of regions with chronic pain, 2) Highest reported intensity in affected regions, and 3) Highest reported bothering in affected regions.

### Selection of covariates

We used a directed acyclic graph (DAG) for selection of covariates to adjust for in the analyses. A DAG is a tool used to minimise bias in analyses, by systematically assessing potential causal relationships between variables [27]. Variables were included in the DAG based on the literature. The DAG was constructed using the software DAGitty [28], which also derived the minimal sufficient adjustment set; sex, age, educational level, generalised sensitivity and worry (S1 Fig).

#### Demographic variables

Age was given as age per December 31, 2015. Participants specified their educational level on a four-point scale; 1) Primary/partly secondary education: up to ten years of schooling, 2) Upper secondary education: a minimum of three years, 3) Tertiary education, short: college/university less than four years, 4) Tertiary education, long: college/university four years or more. Categories 3) and 4) were merged in the analyses.

#### Noise sensitivity as a proxy for generalised sensitivity

Noise sensitivity was used as a proxy for generalised sensitivity, since noise sensitivity has been found to often co-occur with other environmental sensitivities [29]. Noise sensitivity was measured by having participants rate their agreement to item 21 on Weinstein’s Noise Sensitivity Scale; “I am sensitive to noise” [30]. Responses were on a six-point scale ranging from “agree strongly” (1) to “disagree strongly” (6). For analyses, the variable was converted to a three-point scale; 1 (points 4–6), 2 (points 2–3) and 3 (point 1).

This item from the Weinstein Noise Sensitivity Scale was chosen, since it had been used as a one-item measure of noise sensitivity in other studies. We have not found any proper published validation of this one-item measure applied to a general population sample, but a study using a small student sample indicated that although measuring the same underlying construct, the one-item measure was, as would be expected, a more crude measure than more comprehensive measures of noise sensitivity [31]. However, due to lack of space in the Tromsø Study questionnaire, this one-item measure of noise sensitivity from an established instrument was found acceptable to apply instead of a more extensive instrument.

#### Worry

Three central items from the Penn State Worry Questionnaire, as developed by Meyer, Miller, Metzger, and Borkovec (1990) [32], were combined in a single general worry score quantifying the tendency to worry: “I worry all the time”, “Many situations make me worry” and “I am always worrying about something” [33]. Responses ranged from 1 (not at all typical) to 5 (very typical). In accordance with the scoring of these statements in the Penn State Worry Questionnaire [33], response values were summed to one single value for analyses.

The worry items assess the tendency for having uncontrollable chains of thoughts or images that are negatively affect-laden. As somatic health complaints heighten stress-related physiological and cardiovascular activity [34], pain experiences [35], use of medications or health care services in general [36], the Tromsø 7 study included three highly discriminative items from the Penn State Worry Questionnaire. The test score reliability of the Norwegian PSWQ version has been high (Cronbach’s alpha > .90), as well as showing good convergent and discriminative validity [33].

### Statistical analyses

We used multiple logistic regression models to examine the relationships between chronic pain and tinnitus. Tinnitus was set as dependent variable in the analyses. We analysed the association between chronic pain and tinnitus in the full sample (n = 19,039), first using tinnitus >5 minutes as outcome variable, and then applying the stricter tinnitus definitions that included tinnitus frequency and bother. We then analysed the association between each variable describing the chronic pain (number of body regions with chronic pain, highest reported intensity and highest reported bothering) and tinnitus >5 minutes among participants with chronic pain (n = 11,589). Here, the sample was restricted to all participants defined as having chronic pain according to the aforementioned chronic pain definition. We performed these last analyses only for the tinnitus variable >5 minutes, as the similarity of response scales for chronic pain variables and the stricter tinnitus definitions could have produced falsely strong associations between the phenomena.

The regression analyses were adjusted for a minimal covariate set as identified by the DAG. We first ran regression models without any interactions. Then, we tested the interactions between all chronic pain variables and sex, age, noise sensitivity, and worry in separate models, using likelihood-ratio tests, again with tinnitus >5 minutes as outcome variable. It is known that there are differences in prevalence of tinnitus and chronic pain with age and sex [2,37]. We also expected a stronger association among the most sensitive (i.e. those with high score for worry and noise sensitivity). We generated predictive plots for the significant interaction effects, i.e. p-value ≤0.05. The statistical software Stata 15.0 (StataCorp, College Station, TX, USA) was used for all analyses.

## Results

We found an overall tinnitus prevalence (>5 minutes) of 21%, while the overall prevalence of chronic pain was 61%. S1 Table shows tinnitus prevalence according to all four definitions, distributed across the variables included in our models. For all tinnitus definitions, the prevalence of tinnitus was higher among participants with chronic pain than among those without, and more men than women reported having tinnitus. Tinnitus prevalence was lowest in the youngest age group (40–54 years), and prevalence increased with level of noise sensitivity, for all tinnitus definitions. For definitions restricted to at least weekly tinnitus and various degrees of bother, prevalence increased with decreasing level of education. The mean level of worry was higher for participants with tinnitus than for the total sample, and mean worry level increased with increasing tinnitus bother (S1 Table).

**Fig 1:**
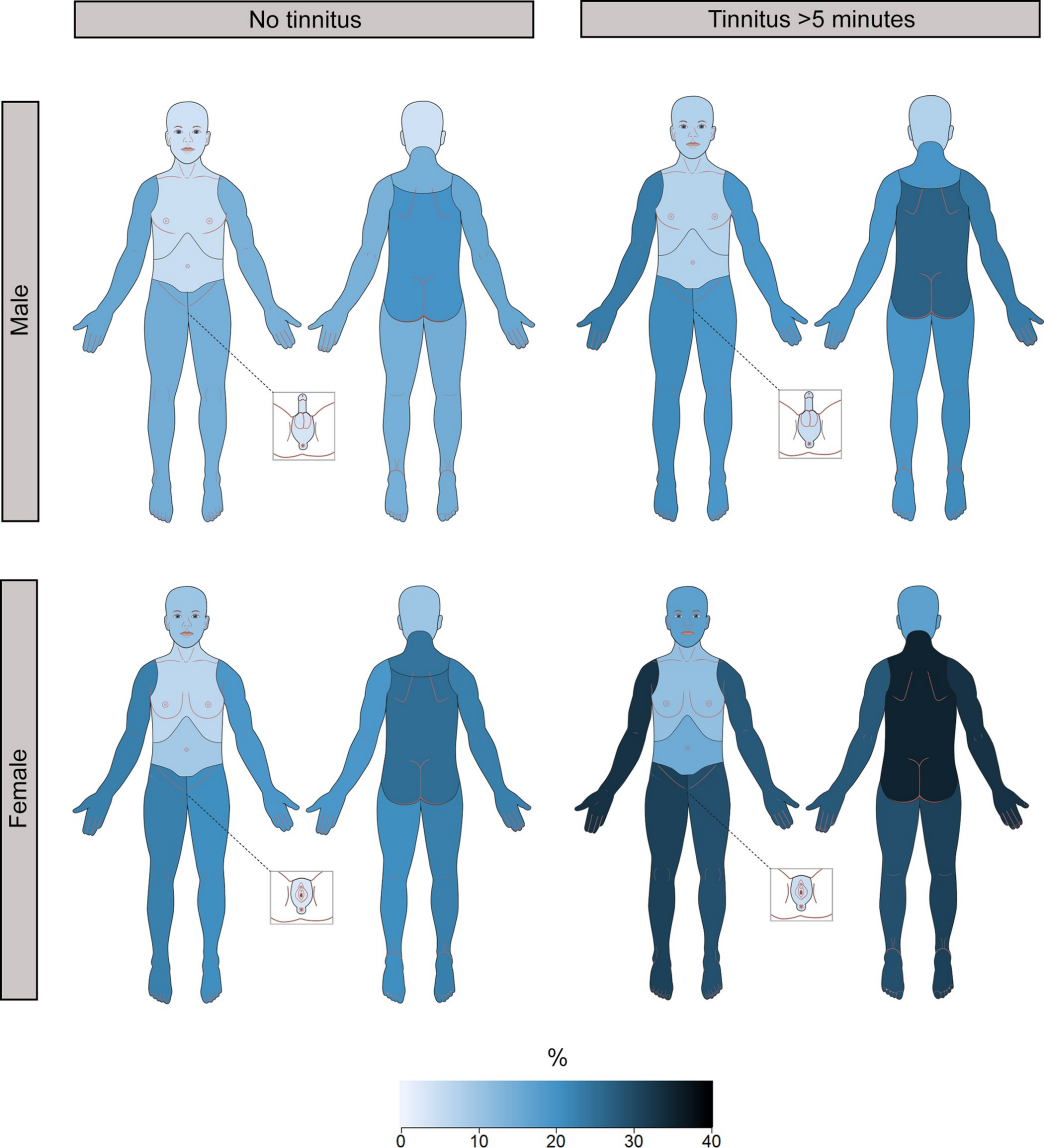
Prevalence and distribution of chronic pain by sex and tinnitus >5 minutes. Darker colour indicates higher prevalence of chronic pain. The Tromsø Study (2015–2016).

Fig 1 shows the prevalence and distribution of chronic pain by sex and tinnitus >5 minutes. Both in individuals with tinnitus and without tinnitus, the prevalence of chronic pain was higher among women than men. Additionally, for both sexes, the prevalence of chronic pain was higher in individuals with tinnitus than in those without. This was true for all ten regions of the body. Thus, men without tinnitus had the lowest prevalence of chronic pain, while women with tinnitus had the highest. For men with tinnitus and women without tinnitus, the prevalence of chronic pain was quite similar. The distribution of chronic pain was similar in all groups, and no particular regions stood out as more affected by chronic pain among individuals with tinnitus.

Table 1 shows the results of the logistic regression analyses of the association between chronic pain and tinnitus, with both broad and stricter tinnitus definitions. The analyses were performed on the full sample (n = 19,039). Odds ratio (OR) for chronic pain gives odds of having tinnitus when having chronic pain, compared to when not having chronic pain. The odds of any tinnitus (>5 minutes) increased by 64% when having chronic pain. The odds were higher with stricter tinnitus definitions–odds of at least weekly and (at least) a little bothersome tinnitus, at least weekly and bothersome tinnitus, and at least weekly and highly bothersome tinnitus, increased by 69%, 95% and 144%, respectively, when having chronic pain.

**Table 1:** Association ^1^ between chronic pain and tinnitus (full sample, n = 19,039).

Independent variable	Tinnitus definition	Model	OR	95% CI	
Chronic pain	Tinnitus >5 minutes	Crude ^2^	1.74***^3^	1.61	1.87
		Full ^4^	1.64***	1.52	1.77
Chronic pain	At least weekly, (at least) a little bothersome tinnitus ^5^	Crude ^2^	1.85***	1.67	2.05
		Full ^4^	1.69***	1.52	1.87
Chronic pain	At least weekly, bothersome tinnitus ^6^	Crude ^2^	2.24***	1.87	2.69
		Full ^4^	1.95***	1.62	2.35
Chronic pain	At least weekly, highly bothersome tinnitus ^7^	Crude ^2^	2.91***	2.06	4.12
		Full ^4^	2.44***	1.72	3.47

The Tromsø Study (2015–2016).

^1^ From multiple logistic regression analyses.

^2^ Adjusted for sex and age.

^3^ p<0.05 was considered statistically significant and is indicated by *. p<0.01 is indicated by **, and p<0.001 is indicated by ***.

^4^ Adjusted for sex, age, educational level, noise sensitivity and worry.

^5^ Tinnitus bother ≥3.

^6^ Tinnitus bother ≥6.

^7^ Tinnitus bother ≥8.

Table 2 shows associations between number of body regions with pain, highest reported pain intensity, highest reported bothering, and tinnitus >5 minutes in participants with chronic pain (n = 11, 589). ORs for number of regions with pain, highest reported pain intensity, and highest reported bothering give the change in odds of having tinnitus >5 minutes for a one unit increase in each chronic pain variable. Thus, with chronic pain in two regions, the odds of having tinnitus >5 minutes increased with 17% (95% confidence interval (CI): 14–20%), compared to when having pain in only one region. With chronic pain in ten regions (maximum effect size), there was a fourfold increase in odds (= 1.167^9 = 4.01). Furthermore, the odds of having tinnitus increased by 6% (95% CI: 4–8%) and 4% (95% CI: 2–7%), respectively, for each one unit increase in pain intensity and bothering. Odds of tinnitus >5 minutes increased by more than 50% comparing maximum pain intensity and bothering to lowest intensity/bothering score (= 1.061^10 = 1.81 / = 1.044^10 = 1.54).

**Table 2:** Associations ^1^ between pain characteristics and tinnitus >5 minutes among participants with chronic pain (n = 11, 589).

Independent variable	Model	OR	95% CI	
Number of body regions with pain	Crude ^2^	1.19***^3^	1.16	1.22
	Full ^4^	1.17***	1.14	1.20
Highest reported pain intensity	Crude ^2^	1.07***	1.05	1.10
	Full ^4^	1.06***	1.04	1.08
Highest reported bothering	Crude ^2^	1.06***	1.04	1.08
	Full ^4^	1.04***	1.02	1.07

The Tromsø Study (2015–2016).

^1^ From multiple logistic regression analyses.

^2^ Adjusted for sex and age.

^3^ p<0.05 was considered statistically significant and is indicated by *. p<0.01 is indicated by **, and p<0.001 is indicated by ***.

^4^ Adjusted for sex, age, educational level, noise sensitivity and worry.

**Fig 2:**
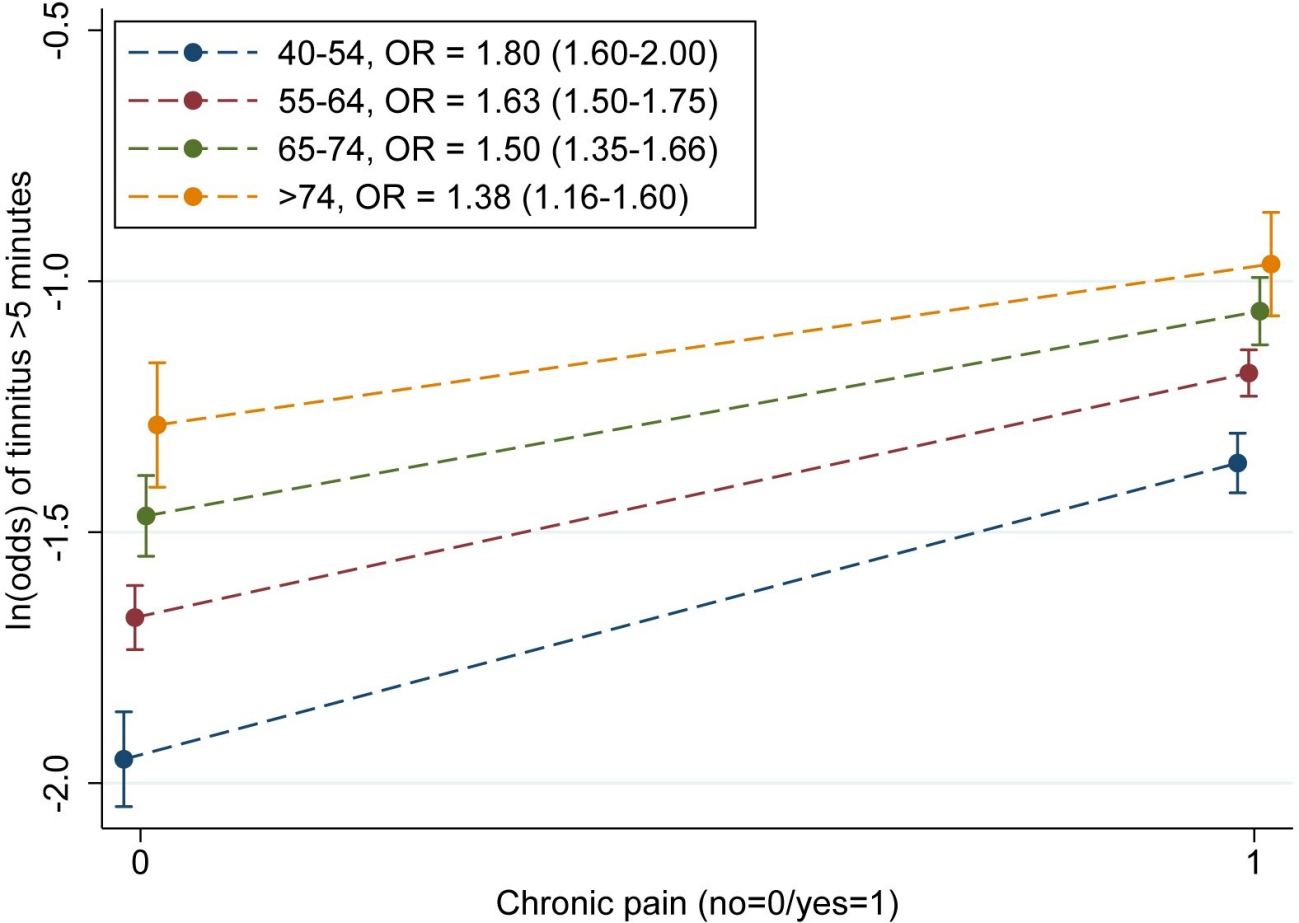
ln(odds) of tinnitus >5 minutes for different age groups, for participants with and without chronic pain. n = 19,039; 95% CIs. The Tromsø Study (2015–2016).

With tinnitus >5 minutes as outcome variable, there were significant interaction effects, on a multiplicative scale, between all chronic pain variables and age (p-values ranging from <0.001 to 0.03). We generated predictive plots showing ln(odds) of tinnitus >5 minutes for different age groups, to illustrate how associations between chronic pain and tinnitus >5 minutes varied with age (Fig 2 and S2–S4 Figs). None of the other interactions tested were significant (S2 Table).

In Fig 2, ln(odds) of tinnitus >5 minutes are illustrated for individuals with and without chronic pain in the different age groups. The association between chronic pain and tinnitus >5 minutes was stronger for younger individuals (OR = 1.80 for age 40–54; i.e. steeper curve) and decreased with age (OR = 1.38 for age >74). Also when graphing ln(odds) for the three separate pain variables in participants with chronic pain (S2–S4 Figs), the association was stronger for the youngest age group for all pain variables, and it weakened with age. Additionally, confidence intervals for age group 40–54 did not overlap with those of the other age groups for the lowest values of chronic pain in any of the cases.

## Discussion

In the present study, participants who reported chronic pain had a higher prevalence of tinnitus than those without, irrespective of tinnitus definition. We found an association between chronic pain and tinnitus that was present for all tinnitus definitions and became stronger when tinnitus was restricted to cases with at least weekly tinnitus and with higher degree of bother. Additionally, among participants with chronic pain, all three chronic pain variables (number of body regions with chronic pain, highest reported pain intensity, and highest reported bothering) were associated with having tinnitus >5 minutes. Of these, the strongest association was found for number of regions with pain. With outcome variable tinnitus >5 minutes, there were significant interactions between all chronic pain variables and age, with the strongest associations for the youngest individuals.

Most of earlier studies focus on specific pain syndromes within specific patient groups. Studies have linked tinnitus to pain syndromes such as headache [4], fibromyalgia [6], and temporomandibular disorder [5]. Additionally, tinnitus prevalence in patients with chronic pain [7] and pain experience in patients with chronic tinnitus [8] have been examined, with results indicating that the two phenomena co-occur among the group of tinnitus and pain patients seeking help in the clinic. However, both tinnitus and chronic pain are broad phenomena, with vast variation in both symptom level and coping. It has been found that only a minority of people with tinnitus consult specialists at audiology clinics [38], and help seekers and non-help seekers may differ in systematic ways [39,40]. To our knowledge, no studies have examined the general relation between tinnitus and chronic pain in the population, and hence, the results of the present study and previous studies are not directly comparable. Nevertheless, the present results align with specific patient group studies indicating that chronic pain and tinnitus are associated phenomena, and add to the knowledge by illustrating that this association exists as a general association with tinnitus broadly defined, but is more pronounced among people with frequent tinnitus.

A study that examined gender-specific differences in comorbidities in individuals with tinnitus found higher rates of chronic shoulder pain and osteoarthritis in both sexes, as well as higher rate of fibromyalgia in women, for individuals with bothersome tinnitus compared to non-bothersome tinnitus [16]. We found the same tendency here–an association between chronic pain and tinnitus that was stronger when the tinnitus definition was restricted to individuals with more bothersome tinnitus.

It has been reported that the association between tinnitus-related distress and pain can be mediated by psychological factors [8]. To examine mediating factors was outside the scope of the present work. However, we did include the variable worry as a covariate, which is closely related to mental health. A high level of general worry is very strongly associated with anxiety of a trait-like (persisting) type, as well as strongly (but more weakly) associated with state-like anxiety. The relationship with depressive symptoms is also high, and on-par with state-like anxiety [33]. According to S1 Table, the level of worry increased when the case definition was restricted to weekly tinnitus. The mean level of worry among tinnitus cases further steadily increased with increasing levels of bother defining the lower limit for counting as a tinnitus case. Since we adjusted for worry in analyses, this variable cannot account for the observed association between chronic pain and tinnitus. However, we recommend that future studies further explore what role psychological factors play in the association between chronic pain and tinnitus.

The fact that we found a stronger association between chronic pain and tinnitus >5 minutes in younger individuals suggests that there could be different mechanisms causing tinnitus and chronic pain in this group than among older individuals. As people age, more individuals are affected by age-related hearing loss, which may also be accompanied by tinnitus [41]. Tinnitus in young subjects may be less related to hearing loss of any kind, including cochlear neuropathy [42]. Tinnitus and chronic pain may thus appear more independently of each other in older age groups, as age-related phenomena. On the other hand, it may be that the strong association between chronic pain and tinnitus >5 minutes found among younger individuals is a result of a common origin of the two phenomena in this group. It may further be speculated that the strongest association is found when tinnitus is not related to hearing loss. It would have been interesting to examine the association between chronic pain and tinnitus also for individuals below the age of 40, to see if the same pattern of association can be found here.

Noise sensitivity was regarded as a good approximation of generalised sensitivity, since noise sensitive individuals have been found to have an accumulation of other sensitivities and symptoms [29]. The inclusion of covariates did not reduce the association between chronic pain and tinnitus substantially, nor was an interaction found for noise sensitivity. Among individuals with chronic pain, the pain variable most strongly associated with tinnitus >5 minutes was number of body regions with pain. Pain in many regions may be related to widespread hyperalgesia, which is often assumed to be linked to central sensitization [43]. This is in accordance with the proposition, based on existing literature [13–15], that chronic pain and tinnitus may be caused by common underlying mechanisms. If our noise sensitivity measure was not a good enough approximation, generalised sensitivity may still be an underlying cause of the association, particularly in young individuals, even though we adjusted for this variable in the analyses. One possible weakness may be that we used a more crude one-item measure of noise sensitivity, instead of a comprehensive instrument. Although residual confounding thus cannot be fully ruled out, it is not likely that a more comprehensive measure of noise sensitivity would have totally changed the conclusions of the study.

Chronic pain was here defined as pain persisting for three months or longer–however, the phrasing of initial GRIP questions caused only participants that had also experienced pain within the past four weeks to be included in the definition. Thus, there is a possibility that some participants with chronic pain were left out, if they had not had pain within the past four weeks. However, when considering the prevalence of chronic pain found here, it is unlikely that our sample omits many participants with chronic pain, since the prevalence was higher in our sample compared with many other studies [26]. Prevalence estimates vary widely across studies, depending on data collection methods and definitions of chronic pain. In the present study, we applied a broad definition of chronic pain (not limited to specific conditions or pain locations) and used different pain characteristics that are sometimes included in the definition of chronic pain, as independent variables in analyses. This allowed us to investigate if some of these characteristics were more strongly associated with tinnitus than others, but may be one explanation of why we found a higher prevalence of chronic pain than many other studies. An additional explanation of the high prevalence found here may be that it was calculated based on many specific questions, rather than a few, more general questions, causing respondents to be reminded of possible pain locations before answering.

Both tinnitus and pain are inherently subjective phenomena. When studying associations between subjectively measured variables, there is a risk of common method bias–that the measurement method systematically affects associations through, for instance, the response style of each participant. This type of bias could create associations between variables that would not exist in real life [44]. However, we have adjusted for several variables in the analyses in order to counter any effects of this bias, and enhance the authenticity of the association.

This study has several strengths. The association between chronic pain and tinnitus has been examined in a large population-based adult sample. The population of Tromsø municipality is representative for the general Norwegian population with respect to age, sex, and educational level [45], and the participation rate in Tromsø 7 was high. Detailed data on pain has been used in this study, as well as several tinnitus definitions–a broad definition most commonly used in population studies (tinnitus >5 minutes), as well as stricter definitions with restrictions on tinnitus frequency and bother. Confounders were identified using DAG, which likely has reduced bias in analyses. We have examined the relationship between chronic pain and a broad tinnitus definition using several different pain variables, and we have tested relevant interaction effects, which, to our knowledge, has not been done before. Our findings are consistent and dose-dependent, with associations between chronic pain and tinnitus that became stronger for at least weekly tinnitus with increasing degree of bother. This strengthens the assumption of an association between the two conditions. A limitation of the study is that we cannot infer anything about temporality in the association between chronic pain and tinnitus, due to the cross-sectional nature of the data. Additionally, our sample did not comprise the entire adult population, since individuals below age 40 were not included. Recommendations for future studies would be to use longitudinal data, which would allow to investigate temporality, and to include individuals below the age of 40, to see if this age group displays a similar pattern of association between chronic pain and tinnitus as what we found in our study.

The association between chronic pain and tinnitus found in our sample stresses the importance of examining for comorbid pain in patients with tinnitus, in order to provide a more comprehensive treatment and prevention of the disorder. In light of these findings, tinnitus treatment methods that overlap with treatment for chronic pain should be considered.

## Supporting information

S1 Fig DAG used for selection of covariates. The minimal adjustment set given by DAGitty was sex, age, educational level, generalised sensitivity and worry.
(PDF)

S2 Fig ln(odds) of tinnitus >5 minutes for different age groups, for each number of body regions with pain, among participants with chronic pain. n = 11,589; 95% CIs. The Tromsø Study (2015–2016).
(TIF)

S3 Fig ln(odds) of tinnitus >5 minutes for different age groups, for each level of pain intensity, among participants with chronic pain. n = 11,589; 95% CIs. The Tromsø Study (2015–2016).
(TIF)

S4 Fig ln(odds) of tinnitus >5 minutes for different age groups, for each level of bothering, among participants with chronic pain. n = 11,589; 95% CIs. The Tromsø Study (2015–2016).
(TIF)

S1 Table Tinnitus prevalence across characteristics of study participants (n = 19,039). The Tromsø Study (2015–2016). ^1^ Tinnitus bother ≥3. ^2^ Tinnitus bother ≥6. ^3^ Tinnitus bother ≥8. ^4^ Defined as pain persisting for 3 months or longer. ^5^ Response to item 21 on Weinstein’s Noise Sensitivity Scale [30]; “I am sensitive to noise”.
(PDF)

S2 Table p-values for interaction effects on tinnitus >5 minutes. (PDF)
