# Supplementary figures and images for: Tinnitus and associations with chronic pain: The population-based Tromsø Study (2015–2016)
Source: PLoS One. 2021 Mar 2;16(3):e0247880. doi: 10.1371/journal.pone.0247880 (PMC7924755; doi:10.1371/journal.pone.0247880)

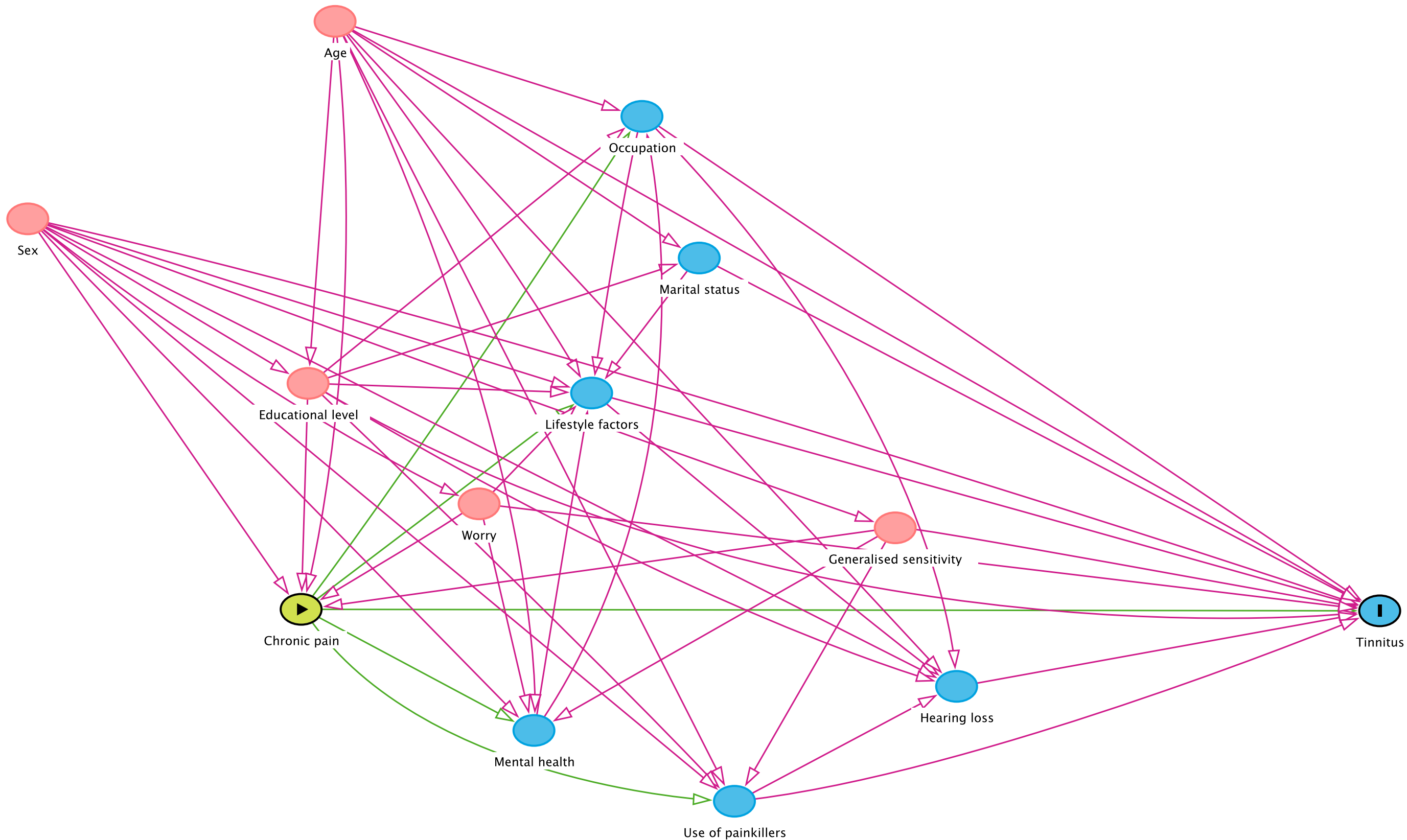

Supplement: S1 Fig — The minimal adjustment set given by DAGitty was sex, age, educational level, generalised sensitivity and worry. (PDF) [file pone.0247880.s001.pdf]

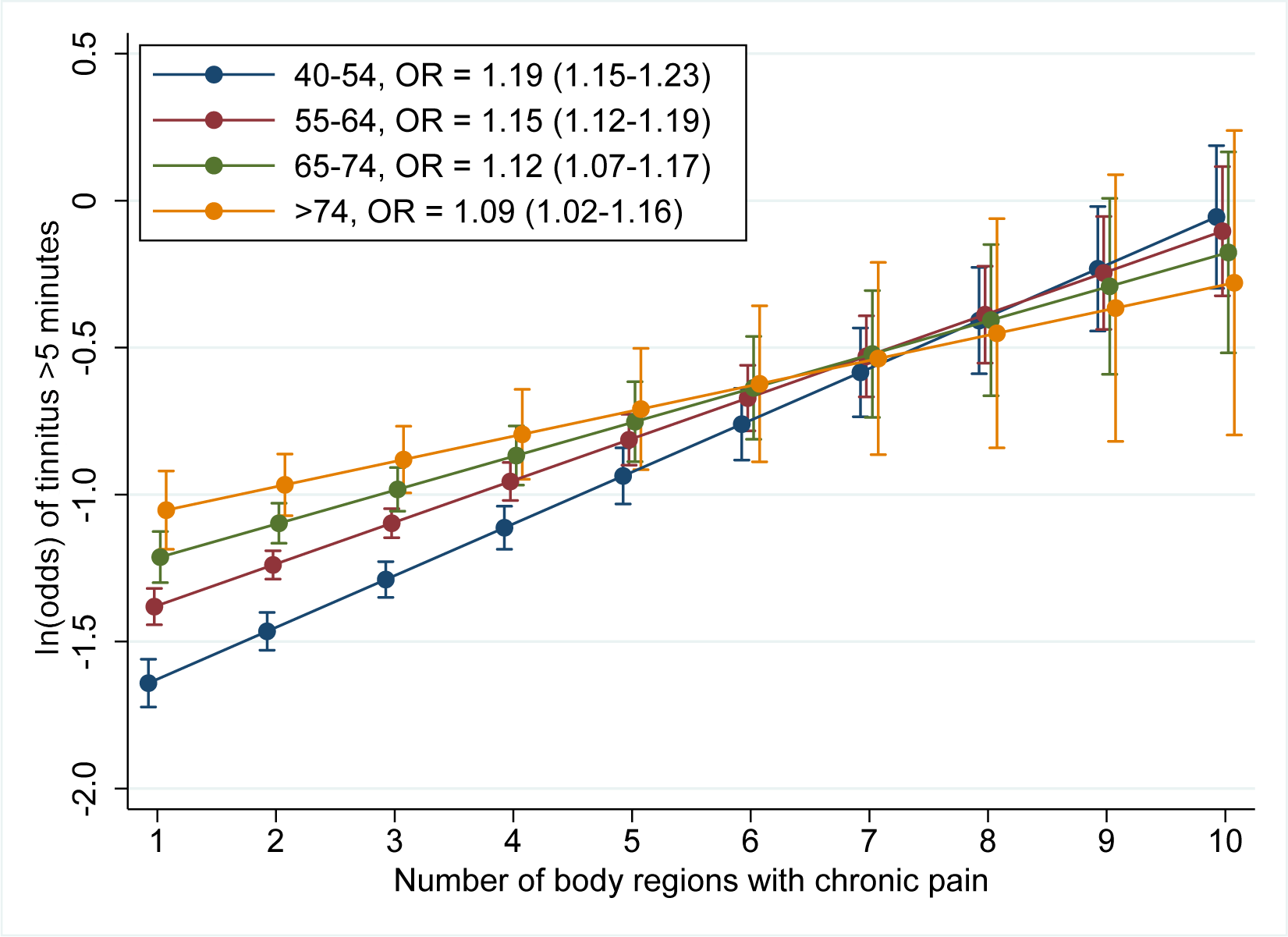

Supplement: S2 Fig — n = 11,589; 95% CIs. The Tromsø Study (2015–2016). (TIF) [file pone.0247880.s002.tif]

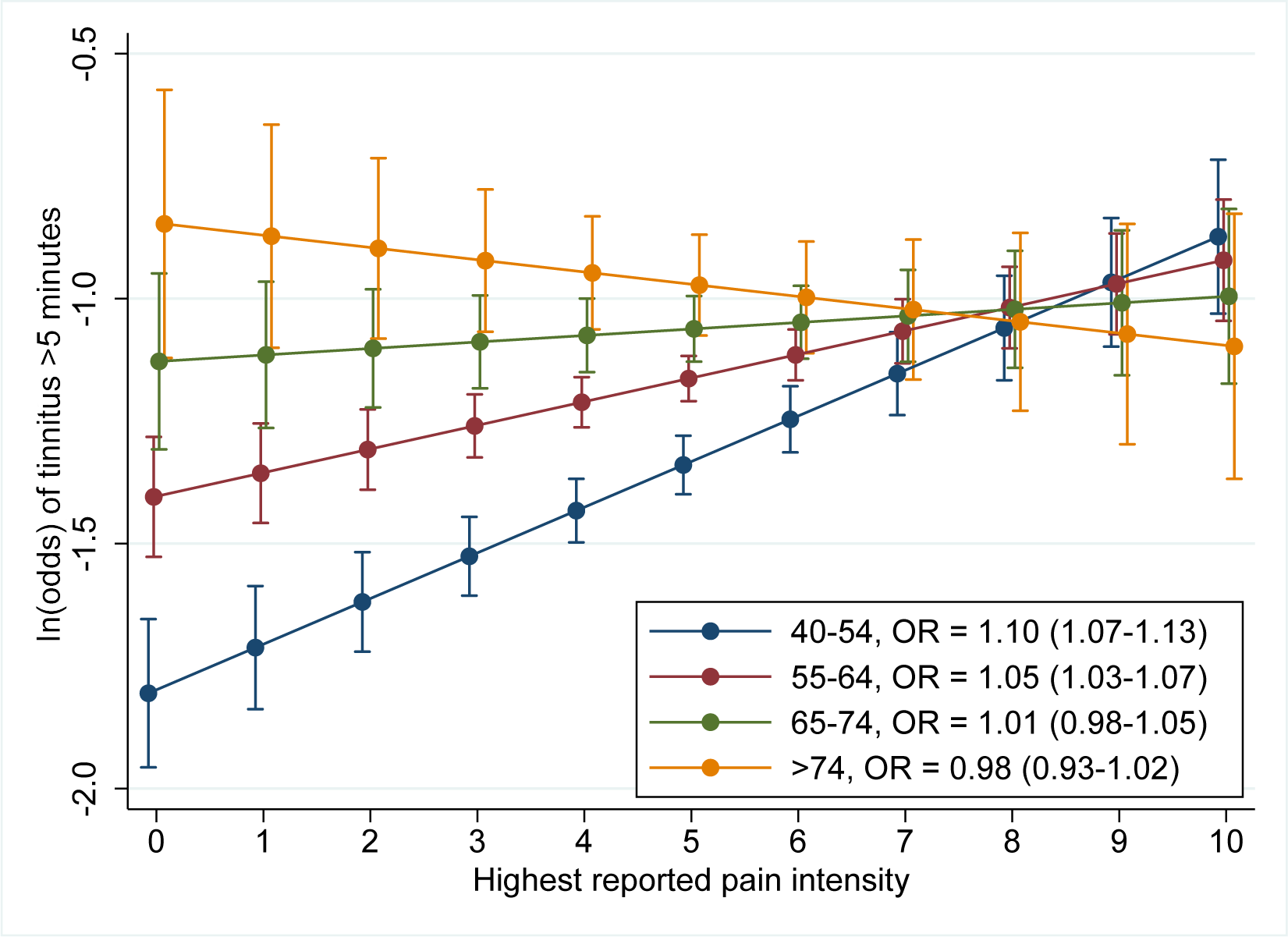

Supplement: S3 Fig — n = 11,589; 95% CIs. The Tromsø Study (2015–2016). (TIF) [file pone.0247880.s003.tif]

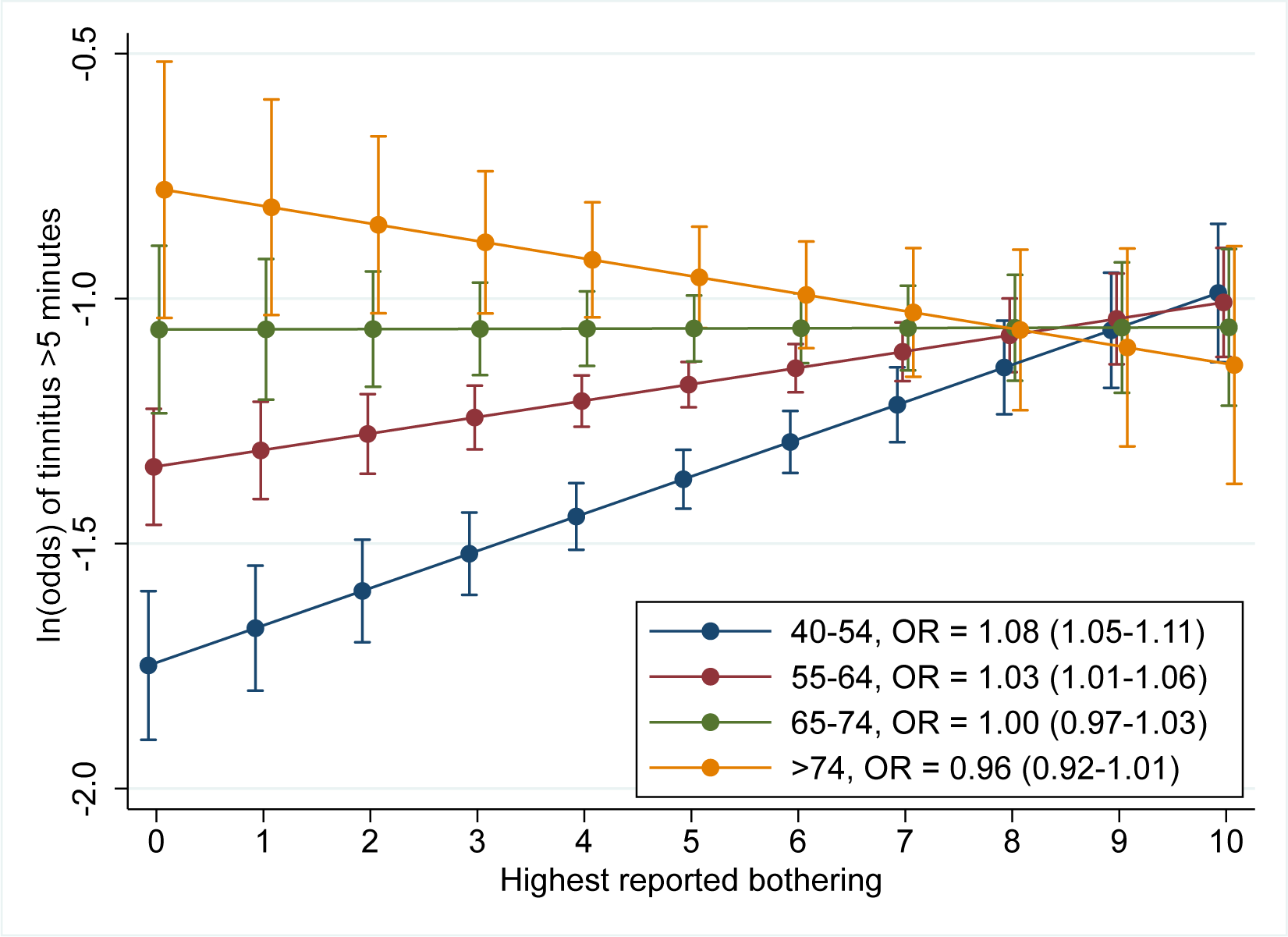

Supplement: S4 Fig — n = 11,589; 95% CIs. The Tromsø Study (2015–2016). (TIF) [file pone.0247880.s004.tif]
