# Supplementary material for: Tinnitus and associations with chronic pain: The population-based Tromsø Study (2015–2016)
Source: PLoS One. 2021 Mar 2;16(3):e0247880. doi: 10.1371/journal.pone.0247880 (PMC7924755; doi:10.1371/journal.pone.0247880)
Supplement: S1 Table — The Tromsø Study (2015–2016). 1 Tinnitus bother ≥3. 2 Tinnitus bother ≥6. 3 Tinnitus bother ≥8. 4 Defined as pain persisting for 3 months or longer. 5 Response to item 21 on Weinstein’s Noise Sensitivity Scale [30]; “I am sensitive to noise”. (PDF) [file pone.0247880.s005.pdf]

	Tinnitus prevalence				
	Tinnitus >5 minutes	At least weekly, (at least) a little bothersome tinnitus [1]	At least weekly, bothersome tinnitus [2]	At least weekly, highly bothersome tinnitus [3]	
	**n (%)**	**n (%)**	**n (%)**	**n (%)**	**Total (100%)**
**Chronic pain [4]**					
No	1 237 (16.6)	581 (7.8)	162 (2.2)	41 (0.6)	7 450
Yes	2 740 (23.6)	1 394 (12.0)	491 (4.2)	161 (1.4)	11 589
**Sex**					
Female	1 604 (16.2)	784 (7.9)	273 (2.8)	84 (0.9)	9 900
Male	2 373 (26.0)	1 191 (13.0)	380 (4.2)	118 (1.3)	9 139
**Age (years)**					
40–54	1 631 (18.2)	696 (7.7)	231 (2.6)	72 (0.8)	8 987
55–64	1 165 (22.6)	601 (11.7)	196 (3.8)	55 (1.1)	5 154
65–74	884 (24.5)	499 (13.8)	161 (4.5)	54 (1.5)	3 615
75 or older	297 (23.2)	179 (14.0)	65 (5.1)	21 (1.6)	1 283
**Education**					
Primary/partly secondary	887 (21.4)	530 (12.8)	213 (5.1)	75 (1.8)	4 140
Upper secondary	1 187 (22.4)	596 (11.2)	213 (4.0)	68 (1.3)	5 310
Tertiary	1 903 (19.9)	849 (8.9)	227 (2.4)	59 (0.6)	9 589
**Noise sensitivity [5]**					
Strongly disagree/disagree/slightly disagree	1 209 (15.8)	507 (6.6)	139 (1.8)	44 (0.6)	7 674
Slightly agree/agree	2 005 (22.2)	998 (11.0)	310 (3.4)	77 (0.9)	9 042
Strongly agree	763 (32.9)	470 (20.2)	204 (8.8)	81 (3.5)	2 323
**Worry (range 3–15) (mean (SD))**	4.8 (2.4)	5.0 (2.6)	5.3 (2.8)	5.5 (3.1)	4.7 (2.3)
**Total**	**3 977 (21.0)**	**1 975 (10.4)**	**653 (3.4)**	**202 (1.1)**	**19 039**
