# Supplementary material for: Tinnitus and associations with chronic pain: The population-based Tromsø Study (2015–2016)
Source: PLoS One. 2021 Mar 2;16(3):e0247880. doi: 10.1371/journal.pone.0247880 (PMC7924755; doi:10.1371/journal.pone.0247880)
Supplement: S2 Table — (PDF) [file pone.0247880.s006.pdf]

		Covariate			
**Independent variable**	n	Sex	Age	Noise sensitivity	Worry
Chronic pain	19,039	p=0.16	p=0.02	p=0.64	p=0.43
Number of body regions with pain	11,589	p=0.93	p=0.03	p=0.56	p=0.26
Highest reported pain intensity	11,589	p=0.82	p<0.001	p=0.65	p=0.49
Highest reported bothering	11,589	p=0.70	p<0.001	p=0.50	p=0.27
